# Pulsed Laser-Bleaching Semiconductor and Photodetector

**DOI:** 10.3390/s24134226

**Published:** 2024-06-29

**Authors:** Chen Huang, Fei Chen, Ze Zhang, Xin Tang, Meng Zhu, Junjie Sun, Yi Chen, Xin Zhang, Jinghua Yu, Yiwen Zhang

**Affiliations:** 1 Changchun Institute of Optics, Fine Mechanics and Physics, Chinese Academy of Sciences, Changchun 130033, China; huangchen22@mails.ucas.ac.cn (C.H.); chenyihit@163.com (Y.C.); zhang315xin@ciomp.ac.cn (X.Z.); yjh10111510@163.com (J.Y.); zhangyiwen@ciomp.ac.cn (Y.Z.); 2 University of Chinese Academy of Sciences, Beijing 100039, China; 3 Aerospace Information Research Institute, Chinese Academy of Sciences, Beijing 100094, China; zhangze@aircas.ac.cn; 4 School of Optics and Photonics, Beijing Institute of Technology, Beijing 100081, China; xintang@bit.edu.cn; 5 No. 8358 Institute of the Third Academy of CASIC, Tianjin 300192, China; zhumeng@tju.edu.cn

**Keywords:** ultrafast dynamics, pulsed laser, semiconductor, photodetector, photonic continuous bleaching

## Abstract

Pulsed lasers alter the optical properties of semiconductors and affect the photoelectric function of the photodetectors significantly, resulting in transient changes known as bleaching. Bleaching has a profound impact on the control and interference of photodetector applications. Experiments using pump–probe techniques have made significant contributions to understanding ultrafast carrier dynamics. However, there are few theoretical studies to the best of our knowledge. Here, carrier dynamic models for semiconductors and photodetectors are established, respectively, employing the rectified carrier drift-diffusion model. The pulsed laser bleaching effect on seven types of semiconductors and photodetectors from visible to long-wave infrared is demonstrated. Additionally, a continuous bleaching method is provided, and the finite-difference time-domain (FDTD) method is used to solve carrier dynamic theory models. Laser parameters for continuous bleaching of semiconductors and photodetectors are calculated. The proposed bleaching model and achieved laser parameters for continuous bleaching are essential for several applications using semiconductor devices, such as infrared detection, biological imaging, and sensing.

## 1. Introduction

Optoelectronic semiconductor devices play important roles in the military, medical, aerospace, and manufacturing industries [1,2,3,4]. The operating bands cover visible to long-wave infrared, depending on the bandgap of semiconductor materials [5,6,7]. When a pulsed laser interacts with semiconductors or semiconductor devices, nonlinear effects are induced, causing a carrier change in the semiconductor [8,9,10,11,12]. Photo-generated electrons transition from the valence band to the conduction band under laser excitation, reducing the optical absorption coefficient of the semiconductor [13]. The carrier relaxation process induced by lasers occurs on a very short time scale, typically ranging from femtoseconds to nanoseconds [14]. This transient effect on the reduced absorption coefficient of the semiconductor is referred to as bleaching [15,16,17]. Therefore, studying the dynamics of carriers induced by pulsed lasers in semiconductors and photodetectors is of significant importance. The pump–probe technique opened new avenues for studying the interaction between pulsed lasers and materials [18]. The first experiment with transient bleaching processes in GaAs semiconductors was achieved. Subsequently, many techniques exploring the ultrafast optical domain have rapidly developed, such as transient absorption spectroscopy [19,20] and transmission or reflection pump–probe detection [21,22]. Many experiments have provided experimental data on pulsed laser-bleaching semiconductors and photodetectors [9,10,17,23,24]. However, the theoretical models remain imperfect. Previous studies by Dou et al. have established bleaching models for direct and indirect bandgap semiconductor materials [25,26], but the photodetector, which is affected by external electromagnetic fields, was not analyzed. Other researchers used the two-temperature model to simulate changes in carrier dynamics and temperature within semiconductors under the action of ultrafast lasers [12,27,28]. Additionally, some have analyzed the temperature response variations in photodetectors [29,30]. Moreover, the analysis was limited to femtosecond or picosecond lasers, and the broadband bleaching model was not realized. To the best of our knowledge, there is no systematic analysis of different laser parameters and different wavelengths to satisfy the requirements of current semiconductors and photodetectors.

In this paper, a carrier dynamics model is established for the interaction of a pulsed laser with a semiconductor and photodetector to simulate the dynamic bleaching effect. The model predicts the temporal variation in carriers in semiconductors and photodetectors composed of silicon, GaAs, Ge, InAs, InSb, HgCdTe, and InAs_0.4_Sb_0.6_ across broad spectrums from visible to long-wave infrared (LWIR). Laser parameters for achieving continuous bleaching of semiconductors and photodetectors are determined by solving the model. Overall, the theoretical model and simulation calculations provide valuable insights for advancements in semiconductor and photodetector technology.

## 2. Materials and Methods

**Figure 1:**
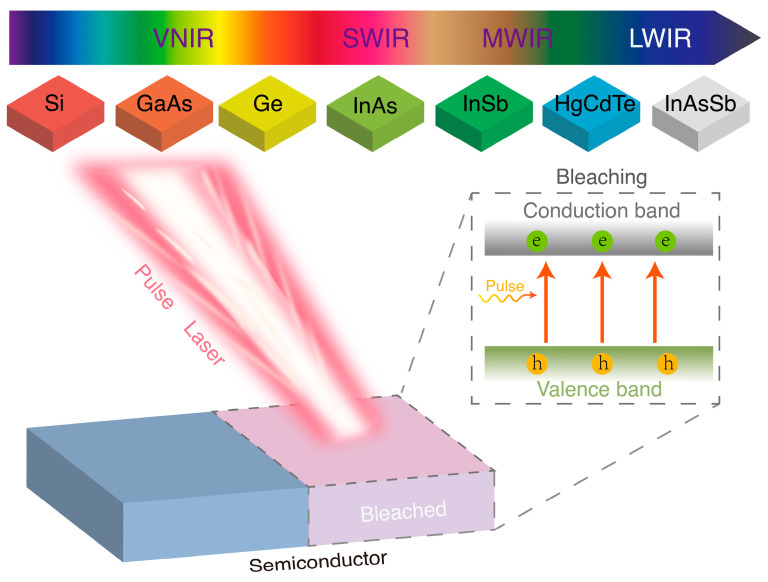
A schematic diagram of the bleaching effect caused by pulsed laser irradiation on a semiconductor, with the coverage range achievable from VNIR (visible and near-infrared) to LWIR (long-wave infrared) wavelengths based on the bandgap of the semiconductor material. The light pink represents the irradiated portion of the laser, while the blue represents the unbleached portion. The diagram inside the dashed box on the right illustrates the principle of pulsed laser bleaching of semiconductors, where photons induce the transition of valence band electrons to the conduction band.

The concept diagram of pulsed laser bleaching of semiconductors is depicted in Figure 1. A short-pulse laser beam excites electrons from the valence band to the conduction band of the semiconductor. The semiconductor’s ability to absorb photons is reduced, which is akin to bleaching. Hence, to achieve continuous bleaching of the semiconductor, one must judiciously choose the laser repetition rate, ensuring that the energy density is adequate to excite all valence electrons while maintaining a pulse duration shorter than the carrier relaxation time. The semiconductor’s bandgap spans from the visible near-infrared (VNIR) to the long-wave infrared (LWIR), allowing for selective excitation of electron transitions within the valence band by selecting appropriate laser wavelengths.

### 2.1. The Model of Pulsed Laser Bleaching Semiconductor

Pulsed laser interactions with semiconductor materials typically involve a sequence of processes, including the excitation of photo-generated carriers, subsequent photon absorption, ionization of the target material, thermalization of carrier–carrier and carrier–phonon scattering, and finally, recombination events such as Auger and radiative recombination [10,11,12,14]. Typically, the processes described involve semiconductors absorbing energy from external sources, which prompts electrons to undergo band-to-band transitions. The spectral absorption coefficient for direct or indirect transitions can be represented in Equation (1) by the following formula [31]:

(1) $$\alpha \left(v\right)=A\sum W_{if}n_{v}(E_{i})n_{c}(E_{f})F(E_{p})$$

where A is the modulation coefficient, $W_{if}$ is the transition probability, $E_{i}$ and $E_{f}$ represent the initial and final energy levels, respectively, $E_{p}$ is the phonon energy, and its probability of action is $F(E_{p})$, distinguishing whether the transition is accompanied by phonons or whether it is an indirect band-to-band transition (for phonon-assisted indirect band-to-band transitions, $F\left(E_{p}\right)=\frac{1}{e^{\left(E_{p}/Tk_{B}\right)}-1}$). $n_{v}$ and $n_{c}$ are the initial state densities of valence band holes and conduction band electrons, respectively, characterizing the ability of the valence band and conduction band to provide holes and electrons. These can be calculated using the initial state density formula given by S. M. Sze’s equation [32]:

(2) $$\left\{\begin{array}{c} n_{v}=2{\left(\frac{2\pi m_{h}^{*}k_{B}T}{h^{2}}\right)}^{\frac{3}{2}}e^{-\frac{E_{F}-E_{i}}{k_{B}T}}\\ n_{c}=2{\left(\frac{2\pi m_{e}^{*}k_{B}T}{h^{2}}\right)}^{\frac{3}{2}}e^{-\frac{E_{f}-E_{F}}{k_{B}T}} \end{array}\right.$$

where $k_{B}=1.38\times 10^{-23}$(J/K) is the Boltzmann constant, $E_{F}$ is the Fermi energy level, $h=6.626\times 10^{-34}$(J·s) is the Planck constant, and $m_{h}^{*}$ and $m_{e}^{*}$ are the effective masses of holes and electrons, respectively (to calculate their values, the electron rest mass $m_{0}$ is utilized here). Due to the low-energy pulsed laser required as the light source for bleaching semiconductor processes and the minimal change in lattice temperature of the target material (this paper assumes a temperature of 300 (K)), the upper limit of carrier concentration that can be excited by the pulsed laser can be represented by the joint density of states in the spectral absorption model [31]:

(3) $$n_{t}=\sqrt{\frac{{\left(2m^{*}\right)}^{\frac{3}{2}}}{2\pi^{2}\hbar^{3}}(E-E_{g})}$$

where the reduced mass of carriers is denoted as $m^{*}=\frac{1}{\frac{1}{m_{e}^{*}}+\frac{1}{m_{h}^{*}}}$. Firstly, it is necessary to consider the relative lengths of the coherence lifetime of coherent states in semiconductors compared to other dynamic processes in the system. If the coherence time of electron–hole pairs in the semiconductor is much shorter than their relaxation time (i.e., the time to reach thermal equilibrium or undergo transitions), then the influence of these coherent effects on the overall absorption and gain behavior can be neglected. This is because coherence is lost within a short time, and the system is mainly controlled by incoherent processes. Under strong pumping conditions, the gain saturation effect becomes significant as a large number of carriers occupy the high energy levels, resulting in a close-to-saturation difference in the population between energy levels. In this scenario, even with additional pump energy, it is not effective in further increasing the number of carriers in the excited state. Hence, the coherent effects associated with pumping become less significant for the overall gain. In certain semiconductor systems, especially those with degenerate or near-degenerate energy-level structures, coherence may naturally be weak, as rapid interconversion between multiple states quickly erases any initial quantum coherence. In establishing theoretical models, disregarding coherent terms aims to simplify the equation set, making it more amenable to analytical solutions or numerical simulations. Then, we can establish a dynamic model suitable for pulsed laser-induced bleaching of semiconductors by integrating models from ultrafast bandgap photonics [9,12,26,33,34]:

(4) $$\left\{\begin{array}{c} \frac{\partial N}{\partial t}=\frac{\alpha \left(v\right)}{E}I-\frac{N-N_{0}}{\tau_{p}}-\frac{N-N_{0}}{\tau_{n}}\\ \frac{\partial P}{\partial t}=\frac{\alpha \left(v\right)}{E}I-\frac{P-P_{0}}{\tau_{p}}-\frac{P-P_{0}}{\tau_{n}} \end{array}\right.$$

where $\tau_{p}$ and $\tau_{n}$ represent the relaxation time of the photo-generated carrier and the carrier lifetime, respectively. $N_{0}$ and $P_{0}$ denote the initial electron and hole concentrations in the semiconductor, and the laser intensity is given by

(5) $$I=\sqrt{\frac{ln2}{\pi }}\frac{1-R}{t_{p}}Je^{-4ln2\frac{{\left(t-t_{p}\right)}^{2}}{t_{p}^{2}}}e^{\alpha \left(v\right)z}$$

Here, $t_{p}$ is the duration of the laser, J is the energy density of the laser, and R is the reflectance. The continuous bleaching process of the semiconductor involves exciting all the ground-state electrons in the semiconductor and maintaining them at the excited level. This process requires the number of self-consuming quantities in the bulk semiconductor to be balanced with the number of photo-generated carriers. Therefore, the duration should be shorter than the relaxation time of the photo-generated carrier. Intrinsic defects in semiconductors create local distortions in the crystal lattice, which can act as scattering centers for phonons. This scattering can enhance the rate of energy loss for excited carriers, leading to faster energy relaxation. Meanwhile, intrinsic defects can also introduce localized electronic states within the bandgap. Carriers can be trapped in these states temporarily before relaxing further, affecting the overall relaxation dynamics. Owing to the plethora of scenarios arising from defects, this paper refrains from delving into an exhaustive analysis thereof. The relaxation rate of the photo-generated carrier can be represented using the model proposed by Gonzalez et al. [35].

(6) $$t_{p}=t_{w,0}+t_{w,1}e^{\left[C1{\left(\frac{T_{n}}{300K}+C0\right)}^{2}+C2{\left(\frac{T_{n}}{300K}+C0\right)}^{2}+C3{\left(\frac{T_{L}}{300K}\right)}^{2}\right]}$$

where $t_{w,0}$, $t_{w,1}$, C0, C1, and C2 are constants that vary with the material. According to the carrier dynamics model derived from Equation (4), the minimum energy density J that excites all ground-state electrons can be deduced for a certain pulse duration.

(7) $$J=\frac{-t_{p}e^{-\alpha \left(v\right)}(\frac{N_{0}-N}{t_{n}}+\frac{N_{0}-N}{t_{p}})}{\hbar v\alpha \left(v\right)\log_{2}\left(\frac{1}{e^{-\frac{E_{f}-E_{c}+Tk_{B}\ln\left(\frac{N}{N_{0}}\right)}{Tk_{B}}}+1}-\frac{1}{e^{-\frac{{E_{v}-E}_{f}+Tk_{B}\ln\left(\frac{N}{N_{0}}\right)}{Tk_{B}}}+1})(R-1\right)}$$

In summary, solving the dynamic model of pulsed laser bleaching of semiconductors can invert the laser parameters inducing continuous bleaching: energy density, pulse duration, and repetition rate.

### 2.2. The Model Pulsed Laser Bleaching Photodetector

**Figure 2:**
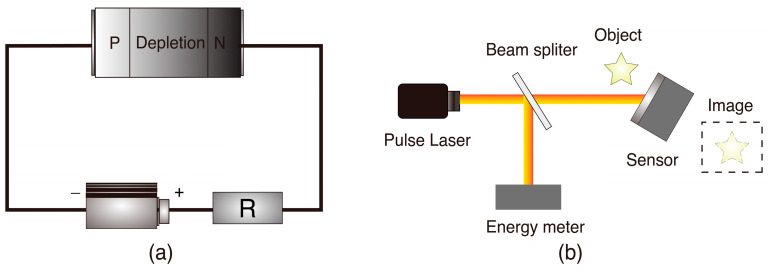
(**a**) The equivalent circuit of a PN junction photodetector consists of equivalent resistances, bias voltage, and the PN junction. (**b**) The ideal setup for a continuous bleaching sensor.

The carrier dynamics during pulsed laser irradiation on a photodetector exhibit distinct characteristics compared to semiconductors, yet they remain interconnected. The photodetector, particularly the photodiode with a composition primarily consisting of semiconductor materials, is central to this process. Utilizing the photoconductive principle, photodiodes generate electron–hole pairs in the depletion region primarily at the PN junction under laser irradiation. Under applied bias voltage, these carriers migrate towards their respective N-type and P-type doping regions, disrupting the equilibrium established by carrier diffusion and drift to produce photocurrent. A typical PN junction with reverse bias, as shown in Figure 2a, consists of a load resistor $R^{\prime }$, bias voltage V, and a closed-loop circuit encompassing the junction, where the PN junction acts as a capacitor. Analysis of this circuit system provides insights into the photodetector’s overall response. In essence, the pulsed laser’s impact on the photodetector can be likened to introducing a charged closed-loop circuit external to the semiconductor. This external bias voltage influences the carrier relaxation time in the conduction band, significantly affecting the recombination velocity of internal carriers.

In the dynamic response of the photodetector to pulsed laser irradiation, the core equations employed are the Poisson equation and the carrier continuity equation [36]. These equations are adapted and modified in Equation (2) to account for the unique characteristics and interactions that occur within the photodetector during laser irradiation.

(8) $$\left\{\begin{array}{c} \frac{\partial n}{\partial t}=\frac{\alpha \left(v\right)}{E}I-R_{Auger}+\frac{1}{q}\nabla \cdot J_{n}\\ \frac{\partial p}{\partial t}=\frac{\alpha \left(v\right)}{E}I-R_{Auger}-\frac{1}{q}\nabla \cdot J_{p}\\ \nabla^{2}\varphi =q(n-p-N_{d}+N_{a}) \end{array}\right.$$

where $N_{d}$ and $N_{a}$ are the ionized donor and acceptor concentrations, ε is the material’s dielectric constant, q is the charge of a single electron, the PN photodetector boasts a self-powered operation capability, and φ is the potential (built-in potential). The equation above, compared to Equation (2), adds the current of generated carriers, $J_{n}$ and $J_{p}$, for electrons and holes, respectively, by Equation (9) and Equation (10).

(9) $$J_{n}=qD_{n}\nabla n-qnV_{n}$$

(10) $$J_{p}=-qD_{p}\nabla p-qpV_{p}$$

where $D_{n\backslash p}$ and $V_{n\backslash p}$ represent the diffusion and drift velocities of carriers, respectively (the subscripts n and p, respectively, represent electrons and holes). The gradient operator in the formula represents differentiation with respect to spatial scale. Their relationship also follows the Einstein distribution $\frac{D}{V}=\frac{kBT}{q}$. Thus, by substituting the existing parameters of the semiconductor into Equation (8), one can obtain the changes in carrier concentration and absorption coefficient. The Auger recombination process [37] is given by $R_{Auger}=\frac{pn-n_{0}^{2}}{t_{n}^{\prime }\left(n+n_{0}\right)+t_{p}^{\prime }(p+p_{0})}$. The calculations of the used laser parameters were derived from semiconductor dynamics inversion.

The continuous laser bleaching phenomenon can also be realized for photodetectors. By solving Equation (8), the appropriate laser pulse energy density is obtained, followed by calculating the modified carrier relaxation time and the velocity at different moments to find a pulse duration and repetition frequency that are both less than this value. An ideal experimental scheme for pulsed laser bleaching of sensors or photodetectors is depicted in Figure 2b. The theoretical calculations outlined previously guide the parameter acquisition for the laser, facilitating its implementation. In the experimental setup, an energy meter is utilized to calibrate the laser parameters. An object is placed in front of the sensor, and upon receiving the appropriately pulsed laser energy, the sensor is rendered ineffective, unable to receive images or parameters of the object. The sustained bleaching effect has been achieved.

## 3. Results and Discussion

**Figure 3:**
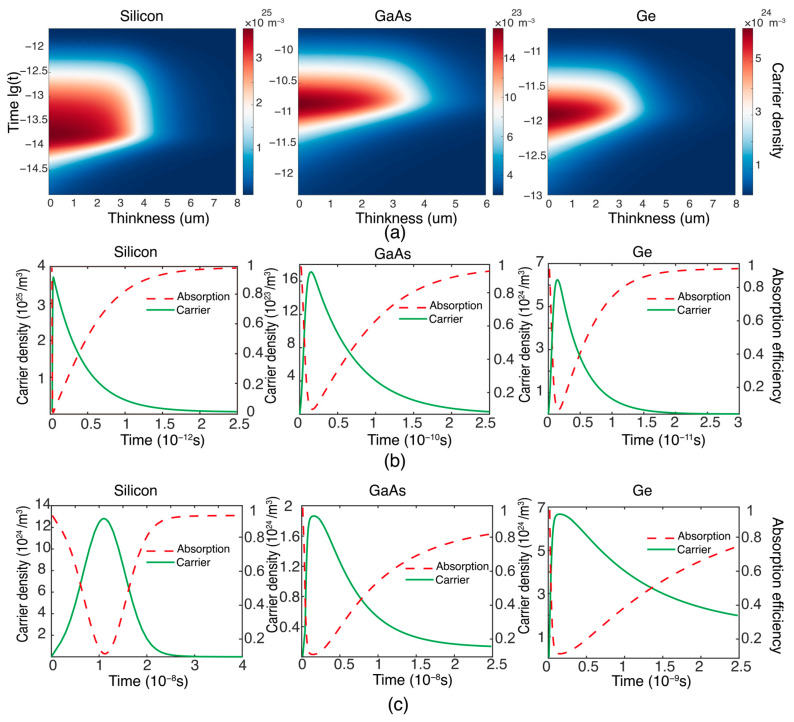
(**a**) Variation in carrier time and spatial scale of semiconductors under VNIR. (**b**) Variation in carrier concentration over time at the semiconductor surface. (**c**) Variation in carrier concentration over time at the photodetector surface. In (**a**–**c**), the materials comprised are silicon, GaAs, and Ge. The color bar in (**a**) represents the carrier concentration, with red dashed lines indicating the absorption coefficient and green solid lines representing the carrier concentration.

In order to validate the feasibility of the aforementioned methods and models, numerical simulations were sequentially conducted across the visible near-infrared (VNIR), short-wave infrared (SWIR), mid-wave infrared (MWIR), and long-wave infrared (LWIR). Furthermore, for the visible light spectrum, correspondence with some existing experimental data serves to uphold the proposed theoretical model. The FDTD (finite-difference time-domain) method is a highly effective approach for solving partial differential equations, which is employed to solve partial differential Equations (4) and (8), with calculations conducted for seven semiconductor and photodetector types: silicon, GaAs, Ge, InAs, InSb, HgCdTe, and InAs_0.4_Sb_0.6_. The parameters of the simulation used are sourced from Table 1 [38,39,40]. Firstly, the effect of laser bleaching on semiconductors in the VNIR is demonstrated in Figure 3a, where the abscissa represents the depth of bleaching and the ordinate denotes the bleaching time. From the figure, it can be observed that when a low-energy, short-pulsed laser acts on the semiconductor, the depth of bleaching is typically at the micron scale. Additionally, the variation over time of surface carrier concentrations in silicon and GaAs, as shown in Figure 3b, conforms to trends observed in existing experiments [41,42]. For instance, within 1 picosecond (ps) in silicon (depicted by the red dashed line), the transient absorption coefficient decreases and carrier concentration increases. The increase in carrier concentration is due to electrons absorbing photon energy, causing them to transition from the ground state to the excited state, or, in other words, from the valence band to the conduction band. This indicates that the semiconductor exhibits absorption characteristics during this process. When the laser excites all the electrons within the semiconductor, the carrier concentration reaches its maximum, and there are no additional electrons available to absorb more light. This results in a decrease in the absorption coefficient, making the semiconductor appear as if it has been bleached. After 2.5 ps, the system returns to equilibrium. It is evident that the laser can influence the absorption coefficient of semiconductor materials. To maintain continuous bleaching, another laser pulse must excite the internal electrons as the carrier concentration decreases back to equilibrium. This repeats the process of electrons absorbing photons and then relaxing. Therefore, the repetition frequency of the laser is $\frac{1}{2.5\;ps}$ the reciprocal of the duration of the entire process. A laser repetition rate of 400 GHz is suggested for bleaching silicon. Solving the partial differential equation leads to Formula (7), from which the laser energy density J required for just sufficient bleaching can be derived as 12 mJ/cm^2^. Laser parameters necessary for other bulk semiconductor bleaching, including energy density, pulse duration, and repetition rate, were inferred and compiled in Table 2. Unlike single semiconductors, photodetectors composed of semiconductor devices consist of circuits and external electronic components, which, as previously described, is tantamount to applying an external electric field to the semiconductor. This external field can affect the device’s response time or the duration that excited electrons remain in the conduction band, thereby absorbing energy. This significantly influences the bleaching mechanism or the parameters required for achieving sustained laser bleaching. Figure 3c illustrates the change in carrier concentration for silicon, GaAs, and Ge photodetectors in the VNIR; their bleaching process resembles that of direct semiconductor action. However, due to the impact on electron relaxation time, the bleaching duration when these components are involved is three orders of magnitude longer compared to the case of bulk semiconductors.

**Figure 4:**
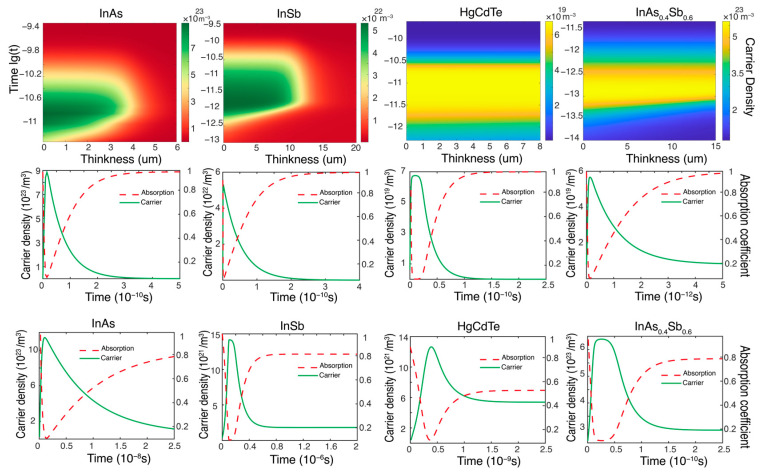
The variations in carrier concentration for InAs, InSb, HgCdTe, and InAs_0.4_Sb_0.6_ semiconductors and detectors in the SWIR, MWIR, and LWIR bands are illustrated. The top row depicts the change in carrier concentration as a function of thickness (abscissa) and time (ordinate). The middle row shows the temporal evolution of surface carrier concentration for bulk semiconductors. The bottom row presents the time response of photodetectors to pulsed laser stimuli.

Different semiconductor materials possess distinct bandgaps, as summarized in Table 1. As the semiconductor band shifts toward longer wavelengths (redshift), the required laser energy decreases accordingly. To investigate the characteristics of carrier concentration changes in SWIR to MWIR semiconductors and detectors, as well as the necessary laser parameters for sustained bleaching, further simulations were conducted on InAs, InSb, HgCdTe, and InAs_0.4_Sb_0.6_ semiconductors and photodetectors. The results are presented in Figure 4. The top row of Figure 4 shows the spatial and temporal variations in carrier concentration. The outcomes for SWIR and MWIR materials InAs and InSb resemble those of visible–near-infrared semiconductors, yet the single-pulse energy densities required for just enough bleaching are 0.17 mJ/cm^2^ and 0.01 mJ/cm^2^, respectively, which are relatively lower compared to the former. Conversely, the corresponding energy densities for LWIR materials HgCdTe and InAs_0.4_Sb_0.6_ are 0.0005 mJ/cm^2^ and 0.1 mJ/cm^2^. Analysis reveals that as the wavelength increases, the bandgap decreases, leading to a reduced number of photons needed to excite electron transitions. From the second row of Figure 4, which displays the surface carrier concentration of the semiconductors, it can be inferred that the relaxation times do not vary significantly. Consequently, the required repetition rate and pulse duration for continuous bleaching are also quite similar, all falling within the picosecond duration and repetition rate of the GHz range. In the last row of Figure 4, the responses of photodetectors made from these four materials to single-pulse laser bleaching are calculated separately, revealing conclusions akin to those observed in the VNIR. All exhibit carrier relaxation times that are shorter than those of bulk semiconductor materials by approximately three orders of magnitude.

**Figure 5:**
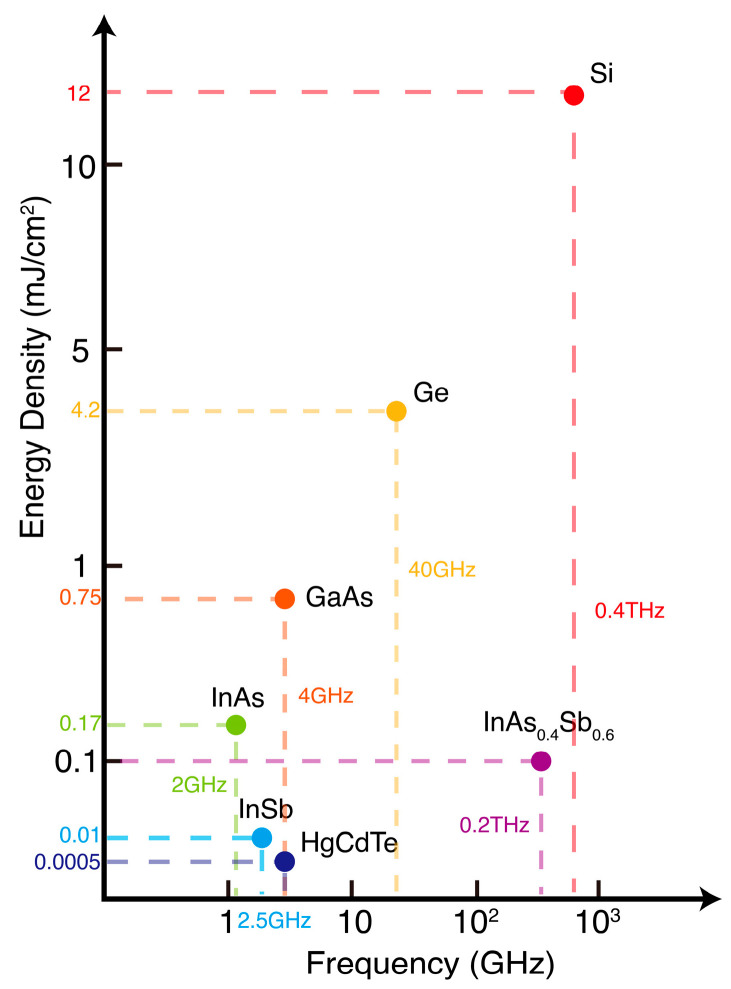
Comparison of laser parameters required for bleaching different semiconductors.

Table 2 and Table 3 outline the laser parameters tailored for continuous bleaching effects, including silicon, GaAs, Ge, InAs, InSb, HgCdTe, and InAs_0.4_Sb_0.6_ semiconductors and photodetectors, respectively. Computational results reveal that the photo-generated carrier relaxation time is significantly longer compared to direct semiconductor analysis. Achieving bleaching in photodetectors allows for a more relaxed repetition rate, typically in the MHz range (orders of magnitude lower than direct semiconductor irradiation), and extended pulse durations up to the nanosecond scale. These findings highlight the unique characteristics of photodetectors for bleaching applications compared to bulk semiconductors. Figure 5 compares the energy density and repetition rate required for bleaching different materials, revealing that the repetition rate is primarily determined by the photo-generated carrier relaxation time, while energy density depends on the joint density of states in the absorption model.

## 4. Conclusions

The mechanism by which a pulsed laser interacts with semiconductors and its device is significant. Internal microscopic particle operation of semiconductors and photodetectors plays a substantial role under pulsed laser excitation. Consequently, this paper has developed a bleaching model that reveals the interaction of a pulsed laser with a semiconductor and a photodetector. The bleaching effect on seven types of semiconductors and photodetectors was comprehensively analyzed and simulated. Materials include silicon, GaAs, Ge, InAs, InSb, HgCdTe, and InAs_0.4_Sb_0.6_, covering broad spectrums from VWIR to LWIR. Furthermore, the FDTD method was employed to solve the model. The laser parameters to induce continuous bleaching in semiconductors and photodetectors were simulated, which meets the real application requirements for broadband operation. This work suggested the bleaching effect in semiconductors and photodetectors induced by pulsed lasers quantitatively, which potentially enables new applications in infrared detection, biological imaging, and sensing.

## Figures and Tables

**Table 1:** Parameters of different semiconductors and photodetectors.

Parameters	Silicon	GaAs	Ge	InAs	InSb	HgCdTe	InAs_0.4_Sb_0.6_
Laser wavelength λ(μm)	0.65	0.65	1.03	2.3	5.4	10	10.4
Band gap Eg (eV)	1.12	1.424	0.661	0.354	0.17	0.123	0.102
$\mathrm{Intrinsic}\;\mathrm{carriers}\;n_{0}$ (m^−3^)	1.5 × 10^16^	2.1 × 10^12^	2 × 10^19^	1 × 10^21^	2 × 10^21^	6.3 × 10^18^	0.8 × 10^23^
Electron effective mass $m_{e}^{*}\;$$(m_{0}$)	1.08	0.063	1.6	0.023	0.014	0.01	0.0104
$\mathrm{Hole}\;\mathrm{effective}\;\mathrm{mass}\;m_{h}^{*}$ $(m_{0}$)	0.57	0.51	0.33	0.41	0.43	0.47	0.422
$\mathrm{Electron}\;\mathrm{lifetime}\;\tau_{n}$ (s)	10^−7^	2.2 × 10^−6^	3.2 × 10^−6^	1.3 × 10^−6^	5 × 10^−8^	10^−6^	9.4 × 10^−7^
$\mathrm{Hole}\;\mathrm{lifetime}\;\tau_{p}$ (s)	10^−7^	2.2 × 10^−6^	3.2 × 10^−6^	1.3 × 10^−6^	5 × 10^−8^	10^−6^	9.4 × 10^−7^
$\mathrm{Carrier}\;\mathrm{energy}\;\mathrm{relaxation}\;\mathrm{time}\;t_{p0}$ (s)	4 × 10^−11^	5.7 × 10^−11^	3.8 × 10^−12^	6.15 × 10^−11^	5 × 10^−11^	2 × 10^−11^	1 × 10^−12^
Refraction n	3.565	3.3	4	3.51	4	3.54	3.7
Reflection R	0.5	0.46	0.722	0.54	0.415	0.11	0.126
$\mathrm{Acceptor}\;\mathrm{density}\;N_{a}$ (m^−3^)	2 × 10^23^	0.3 × 10^23^	6.3 × 10^22^	2 × 10^23^	7.7 × 10^20^	1.5 × 10^21^	2.5 × 10^23^
$\mathrm{Donor}\;\mathrm{density}\;N_{d}$ (m^−3^)	4.9 × 10^21^	1.2 × 10^23^	0.8 × 10^22^	1 × 10^23^	3.85 × 10^20^	1 × 10^21^	1.5 × 10^23^
$\mathrm{Mobility}\;\mathrm{velocity}\;\mathrm{of}\;\mathrm{the}\;\mathrm{electron}\;V_{n}$ (cm^2^·V^−1^·s^−1^)	1400	8500	3900	40,000	7.7 × 10^4^	1 × 10^5^	2.25 × 10^4^
$\mathrm{Mobility}\;\mathrm{velocity}\;\mathrm{of}\;\mathrm{hole}\;V_{p}$ (cm^2^·V^−1^·s^−1^)	450	400	1900	500	850	450	500

**Table 2:** Parameters of laser parameters for pulsed laser continuous bleaching of semiconductors.

Laser Parameters	Silicon	GaAs	Ge	InAs	InSb	HgCdTe	InAs_0.4_Sb_0.6_
Energy density (mJ/cm^2^)	12	0.75	4.2	0.17	0.01	0.0005	0.1
Pulse duration (ps)	0.01	10	1	10	1	10	0.1
Repetition rate (GHz)	400	4	40	2	2.5	4	200

**Table 3:** Parameters of laser parameters for pulsed laser continuous bleaching of photodetectors.

Laser Parameters	Silicon	GaAs	Ge	InAs	InSb	HgCdTe	InAs_0.4_Sb_0.6_
Energy density (mJ/cm^2^)	13	1.7	3	0.15	0.05	0.005	0.05
Pulse duration (ps)	10,000	1000	100	1000	100,000	100	10
Repetition rate (MHz)	25	40	400	40	2	400	4000

## Data Availability

The data presented in this study are available on request from the corresponding author.

## References

[1] Camposeo A., Persano L., Farsari M., Pisignano D. (2019). Additive manufacturing: Applications and directions in photonics and optoelectronics. Adv. Opt. Mater..

[2] Hegazy M.A., Abd El-Hameed A.M. (2014). Characterization of CdSe-nanocrystals used in semiconductors for aerospace applications: Production and optical properties. NRIAG J. Astron. Geophys..

[3] Maini A.K. (2010). Battlefield Lasers and Opto-electronics Systems. Def. Sci. J..

[4] Walkey C., Sykes E.A., Chan W.C. (2009). Application of semiconductor and metal nanostructures in biology and medicine. ASH Educ. Program Book.

[5] González A., Fang Z., Socarras Y., Serrat J., Vázquez D., Xu J., López A.M. (2016). Pedestrian detection at day/night time with visible and FIR cameras: A comparison. Sensors.

[6] Piprek J. (2013). Semiconductor Optoelectronic Devices: Introduction to Physics and Simulation.

[7] Velicu S., Grein C., Emelie P., Itsuno A., Philips J., Wijewarnasuriya P. (2010). MWIR and LWIR HgCdTe infrared detectors operated with reduced cooling requirements. J. Electron. Mater..

[8] Bendib A., Bendib-Kalache K., Deutsch C. (2013). Optical breakdown threshold in fused silica with femtosecond laser pulses. Laser Part. Beams.

[9] Di Cicco A., Polzoni G., Gunnella R., Trapananti A., Minicucci M., Rezvani S., Catone D., Di Mario L., Pelli Cresi J., Turchini S. (2020). Broadband optical ultrafast reflectivity of Si, Ge and GaAs. Sci. Rep..

[10] Guo B., Sun J., Lu Y., Jiang L. (2019). Ultrafast dynamics observation during femtosecond laser-material interaction. Int. J. Extrem. Manuf..

[11] Othonos A. (1998). Probing ultrafast carrier and phonon dynamics in semiconductors. J. Appl. Phys..

[12] Zhang F., Li S., Chen A., Jiang Y., Li S., Jin M. (2016). Ultrafast dynamical process of Ge irradiated by the femtosecond laser pulses. High Power Laser Sci. Eng..

[13] Klingshirn C.F. (2012). Semiconductor Optics.

[14] Sundaram S., Mazur E. (2002). Inducing and probing non-thermal transitions in semiconductors using femtosecond laser pulses. Nat. Mater..

[15] Rafailov M.K. (2011). Ultrafast bandgap photonics. Micro-and Nanotechnology Sensors, Systems, and Applications III.

[16] Rafailov M.K. (2016). Ultrafast bandgap photonics: Meta-stability of transient states. Ultrafast Bandgap Photonics.

[17] Xu Z., Zhang J., Lin X., Shao B., Yang P. (2017). Negative response of hgcdte photodiode induced by nanosecond laser pulse. Proceedings of the Fourth International Symposium on Laser Interaction with Matter.

[18] Zewail A.H. (1988). Laser femtochemistry. Science.

[19] Niedzwiedzki D.M., Sullivan J.O., Polívka T., Birge R.R., Frank H.A. (2006). Femtosecond time-resolved transient absorption spectroscopy of xanthophylls. J. Phys. Chem. B.

[20] Ohkita H., Cook S., Astuti Y., Duffy W., Tierney S., Zhang W., Heeney M., McCulloch I., Nelson J., Bradley D.D. (2008). Charge carrier formation in polythiophene/fullerene blend films studied by transient absorption spectroscopy. J. Am. Chem. Soc..

[21] Hernandez-Rueda J., Puerto D., Siegel J., Galvan-Sosa M., Solis J. (2012). Plasma dynamics and structural modifications induced by femtosecond laser pulses in quartz. Appl. Surf. Sci..

[22] Pan C., Wang Q., Sun J., Wang F., Sun J., Wang G., Lu Y., Jiang L. (2019). Dynamics and its modulation of laser-induced plasma and shockwave in femtosecond double-pulse ablation of silicon. Appl. Phys. Express.

[23] Wang K., Yao C., Wu Y., Wang X., Wang Y., Li P. (2023). Laser-interfered studies in HgCdTe infrared focal plane array detector by high-repetition-rate mid-infrared supercontinuum fiber laser. Opt. Laser Technol..

[24] Zuodong X., Jianmin Z., Xinwei L., Bibo S. (2018). Transient response degradation of HgCdTe photovoltaic detectors under irradiation of nanosecond laser. Infrared Laser Eng..

[25] Dou X., Sun X. (2012). Model of transient bleaching effect of the direct bandgap semiconductor induced by femtosecond laser. Zhongguo Jiguang(Chin. J. Lasers).

[26] Dou X., Sun X., Li H., Chen X. (2015). The study of transient bleaching effect of indirect bandgap semiconductors induced by femtosecond laser. Optik.

[27] Abdelmalek A., Kotsedi L., Bedrane Z., Amara E.-H., Girolami M., Maaza M. (2022). Optical and thermal behavior of germanium thin films under femtosecond laser irradiation. Nanomaterials.

[28] Principi E., Giangrisostomi E., Mincigrucci R., Beye M., Kurdi G., Cucini R., Gessini A., Bencivenga F., Masciovecchio C. (2018). Extreme ultraviolet probing of nonequilibrium dynamics in high energy density germanium. Phys. Rev. B.

[29] Du L., Sun J., Zhang R. (2013). Transient response analysis of photoconductive detector under ultra-short laser pulse radiation. Optik.

[30] Meng Q., Yu J., Zhong Z., Ye R., Zhang B. (2015). Damage threshold prediction of crystal materials irradiated by femtosecond lasers based on ionization model and two-temperature model. Opt. Mater..

[31] Kuzmany H. (2009). Solid-State Spectroscopy: An Introduction.

[32] Green M.A. (1990). Intrinsic concentration, effective densities of states, and effective mass in silicon. J. Appl. Phys..

[33] Li M., Menon S., Nibarger J.P., Gibson G.N. (1999). Ultrafast electron dynamics in femtosecond optical breakdown of dielectrics. Phys. Rev. Lett..

[34] Tanimura H., Kanasaki J.i., Tanimura K., Sjakste J., Vast N. (2019). Ultrafast relaxation dynamics of highly excited hot electrons in silicon. Phys. Rev. B.

[35] Gonzalez B., Palankovski V., Kosina H., Hernandez A., Selberherr S. (1999). An energy relaxation time model for device simulation. Solid-State Electron..

[36] Selberherr S. (2012). Analysis and Simulation of Semiconductor Devices.

[37] Zhang Y., Dou X., Li F., Sun X. (2015). The response characteristics of avalanche photodiodes to ultrashort pulsed laser. Infrared Phys. Technol..

[38] Chuang S.L. (2012). Physics of Photonic Devices.

[39] Kopytko M., Sobieski J., Gawron W., Kębłowski A., Piotrowski J. (2021). Minority carrier lifetime in HgCdTe (100) epilayers and their potential application to background radiation limited MWIR photodiodes. Semicond. Sci. Technol..

[40] Levinshtein M. (1997). Handbook Series on Semiconductor Parameters.

[41] Feng T., Chen G., Han H., Qiao J. (2021). Femtosecond-laser-ablation dynamics in silicon revealed by transient reflectivity change. Micromachines.

[42] Turchinovich D., D’Angelo F., Bonn M. (2017). Femtosecond-timescale buildup of electron mobility in GaAs observed via ultrabroadband transient terahertz spectroscopy. Appl. Phys. Lett..

